# The role of body composition in neurological and hematologic toxicity in a retrospective analysis of 120 breast cancer patients undergoing neoadjuvant chemotherapy: the COMBOTOX study

**DOI:** 10.1007/s10549-024-07553-x

**Published:** 2024-12-04

**Authors:** A. Di Leone, A. Filippone, C. Maggiore, M. M. Rossi, C. Rossi, A. Di Micco, Luana Forcina, A. Franco, L. Ionta, A. Fabi, I. Paris, L. Scardina, A. M. Sanchez, P. C. Pafundi, G. Franceschini, R. Masetti, S. Magno

**Affiliations:** 1 Breast Unit, Department of Women, Children and Public Health Sciences, Fondazione Policlinico Universitario “A. Gemelli” IRCCS, Largo Agostino Gemelli, 8, 00136 Rome, Italy; 2 Center for Integrative Oncology, Fondazione Policlinico Universitario A. Gemelli IRCCS, Rome, Italy; 3 Precision Medicine Breast Unit, Scientific Directorate, Department of Women, Children and Public Health Sciences, Fondazione Policlinico Univesitario “A. Gemelli” IRCCS, Rome, Italy; 4 Department of Women, Children and Public Health Sciences, Fondazione Policlinico Univesitario “A. Gemelli” IRCCS, Rome, Italy; 5 Epidemiology & Biostatistics Research Core Facility, Gemelli Generator, Fondazione Policlinico Universitario Agostino Gemelli IRCCS, Rome, Italy

**Keywords:** Breast cancer, Neoadjuvant chemotherapy, Body composition, Toxicity, Personalized medicine

## Abstract

**Purpose:**

Neoadjuvant chemotherapy (NAC) has a well-established role in locally advanced or chemoresponsive breast cancers (BC). Chemotherapic regimens are effective when patients receive the optimal doses. Toxicities are common in overweight/obese patients but may occur also in normal weight counterparts. This leads to delays, reductions, or discontinuation of treatment, with impact on outcomes. Current dosing is based on body weight and predicted Body Surface Area (BSA). These parameters do not take into consideration the individual variations of fat mass (FM) and fat-free mass (FFM) that affect pharmacokinetics. Assessment of body composition (BoCo), rather than Body Mass Index (BMI), could help to better plan chemotherapy and reduce drug-related toxicities. Our aim was to analyze the correlations between body weight, anthropometric measures, BoCO, and toxicities related to NAC in non-metastatic BC patients.

**Methods:**

This is a retrospective observational cohort study that includes 120 consecutive BC patients undergoing NAC, enrolled between May 2018 and December 2020. All patients received an evaluation of anthropometric parameters (height, weight, waist and hip circumference, BMI) and an assessment of BoCo using Segmental Multi-Frequency-Bioelectrical Impedance Analysis.

**Results:**

A logistic regression models confirmed that a higher FM was associated with a higher rate of neurological and hematologic toxicities in protocols containing Platinum. Moreover, patients with a low FFM% have a higher risk for hematologic toxicity in protocols containing Platinum.

**Conclusion:**

A routine assessment of BoCo, in addition to evaluation of anthropometric measures and BMI, could allow to personalize chemotherapy doses, in order to reduce chemotherapy-related toxicities.

## Introduction

Neoadjuvant chemotherapy (NAC) is a cornerstone of BC treatment in locally advanced disease, but toxicities, delays, and discontinuation of the scheduled regimens remain a major concern [1–7]. Patients’ body weight has remarkable effects on chemotherapy-induced toxicity [8], leading to more common dose reductions in obese patients [9, 10], particularly in high-dose density regimens [11].

Toxicities may occur also in normal weight patients, due to a possible abnormal distribution of fat mass and other body components (muscle mass, bone mass, and body water) [12].

Chemotherapy drugs are traditionally dosed according to the predicted body surface area (BSA); therefore patients with higher body mass index (BMI) receive higher doses of drugs even if it is known that patients with a similar BSA/BMI may have wide variations in the amount and distribution of fat mass (FM) and fat-free mass (FFM) and that these parameters may influence the pharmacokinetics of chemotherapic agents and potentially increase toxicities and worsen cancer treatment outcomes [13–15].

In fact, the study of Bradshaw and other reviews in the Literature underline that up to 40% of obese cancer patients cannot maintain the full dose of chemotherapy during their entire course of treatment because of significant toxicities and suggest that this may explain the higher mortality rates observed in this subset of patients [16].

Nonetheless, the 2021 update of the American Society of Clinical Oncology (ASCO) guidelines on the appropriate systemic therapy dosing for obese adult patients recommends the use of full-weight-based cytotoxic chemotherapy doses in obese patients. The importance of more customized dosing, based on pharmacokinetic and pharmacogenetic factors, sarcopenia, and other body composition parameters are viewed by the ASCO panel only as areas for future research [17].

The aim of this retrospective study was to investigate the correlations between body weight, body composition, and toxicities/adverse events in BC patients receiving NAC.

## Methods

### Study design

This is a retrospective observational pilot cohort study, performed on 120 consecutive patients who underwent neoadjuvant chemotherapy for non-metastatic breast cancer at Policlinico Universitario Agostino Gemelli IRCCS in Rome (Italy) from May 1st 2018 to December 31th 2020. The study is compliant with 1976 Declaration of Helsinki and its later amendments and received formal approval by the local Ethics Committee (n. 5357, retrospectively registered). Provided a signed written informed consent, data were retrieved from the medical charts and reported in an electronic database.

All the patients were screened for anthropometric and BoCo measures as described in the following paragraphs. Anthropometric measures included height, weight, BMI, waist circumference, and waist hip ratio (WHR). Height was measured to the nearest 0.5 cm using a mechanical stadiometer with the patient in standing position and without shoes, facing directly ahead, feet together, arms by the sides, heels, buttocks, and upper back in contact with the stadiometer. Weight was measured to the nearest 0.1 kg, without clothes, using a mechanical scale accurate. BMI was calculated using the following formula: kg/m^2^. Circumferences were measured at the smallest circumference of the waist, above the belly button, at widest part of the buttocks (hip). Every measure was approximated to the nearest 0.5 cm using a stretch-resistant tape (Ergonomic circumference measuring tape; SECA 201; GmbH & Co. KG, Hamburg, Germany). Waist–hip ratio was calculated using the following formula: waist circumference (cm)/hip circumference (cm).

Body composition assessment was performed using segmental multi-frequency-bioelectrical impedance analysis (SMF-BIA; DS Medical model Human in touch; Milan, Italy), a safe and easy procedure routinely employed in our clinical practice [18]. Physical parameters directly measured in Ohms were resistance (Z), reactance (Xc) at 50 kHz, and 800-μA phase angle (Φ) calculated using the following equation: phase angle = (resistance/reactance) × (180/π). Derived parameters considered were fat mass (FM) and fat-free mass (FFM) both in kilograms (kg) and percentage (%). Hydration referred to total body water (TBW) was calculated in liters (L) and percentage (%).

As regards the histopathological features, consistent with the immunohistochemical findings, the following subgroups of clinical relevance were identified: Luminal B/HER2 negative (Type B)—hormone receptor positive, HER2 negative, Luminal B/HER2 positive (Type BH)—hormone receptor positive, HER2 positive, Her2 positive (Type H) non-luminal with overexpressed HER2, and Triple negative (TN)—HER2 negative, no hormone receptor expression.

As regards the chemotherapy regimens, the majority of patients (77; 64.2%) received anthracycline-taxane-based protocols (Th1 regimen), while in the remaining patients anthracycline-taxanes plus carboplatin were administered (Th2 regimen).

### Endpoints

The aim of this study was to investigate the association between body weight, body composition assessed by Bioelectrical impedance analysis (BIA), and the main chemo-related toxicities in BC patients receiving NAC. Among the secondary outcomes, we aimed to evaluate (i) whether a higher fat component would be the leading cause of chemotherapy interruption and (ii) potential correlations with common side effects (including hypertransaminasemia, hot flashes, headache, constipation, epigastralgia, onychopathy, osteoarticular pain, asthenia, diarrhea, emesis, and mucositis).

### Statistical analysis

Due to the paucity of clinical studies about this topic, this work was conceived as a pilot study and, consequently, did not require any formal sample size calculation. However, the total sample (120 patients) is consistent with the common rule of thumb for pilot studies by Sim and Lewis (2011), [19] that foresees a minimum sample size of 55 subjects for small to medium effect sizes. Indeed, due to the retrospective nature of the study, a power calculation demonstrated that a sample size of 120 patients would achieve 90.8% power to reject the null hypothesis of zero effect size when the population effect size is 0.30 and the significance level (alpha) is 0.050 using a two-sided one-sample z test. Power calculation was performed with PASS2021 software (NCCS Statistical Software) [https://www.ncss.com/].

All data were summarized by descriptive statistics techniques. In depth, qualitative data were expressed as absolute and percentage frequencies, while quantitative variables either as mean and standard deviation (SD) or median and interquartile range (IQR). Gaussian distribution was assessed by the Shapiro–Wilk’s test.

In order to evaluate the raw effects of each body composition predictor on toxicity due to chemotherapy, logistic regression models were fitted. The exponential transformation of the regression coefficients associated to the predictor allowed more easily interpretable odds ratios (ORs), i.e., the odd variation per unit increase of predictor. To evaluate the potential combined effects between predictors and type of chemotherapy administered, multivariable interaction logistic regression models were fitted, and the interaction odd ratios (IOR) were evaluated. In particular, for each predictor, one interaction model was fitted. In this framework, IOR = 1 indicated no synergy between predictor and therapies different from the most common (i.e., Th1), IOR < 1 expressed a reduction of odd due to the synergy, while IOR > 1 denoted an increased odd. In summary, the coefficients of the main effects (in exponential terms) were interpreted particularly as ORs of the outcome by considering a unit increase of the predictor marker in the Th1 case (ORpredictor). The interaction parameters were interpreted as difference (in OR terms) of the predictor variations between therapeutic regimens conditions (Th1 as reference category). The same models were also applied to assess the role of fat component on chemotherapy interruption.

To evaluate the potential combined effects between predictors and type of chemotherapy administered, multinomial interaction regression models were fitted, and the interaction risk ratios (IRR) evaluated, as aforementioned.

Statistical significance was set at *P* value < 0.05. *P* values between 0.05 and 0.10 were also reported as suggestive. All analyses were performed using R software Version 4.1.2 (CRAN ®, R Core Team, 2021) and “nnet” package [https://www.r-project.org/].

## Results

### General characteristics of the study population

Women enrolled in the study had a mean age of 49.3 years (SD 11.9) and a median BMI of 25 kg/m2. Almost half of the study population was in menopausal status (n = 54; 45%) before starting the chemotherapic protocols.

Waist and hip circumferences were within the reference ranges (median 88 cm and 102 cm, respectively), with a mean Waist–Hip Ratio (WHR) of 0.87.

As regards body composition, we observed a mean FM% of 32.7% (SD 8.5) and a median FFM of 67.5% that are, respectively, above and under the standard cut-off for healthy adult women.

The BC subtypes were homogeneously distributed, with the “H” type as the least prevalent (11; 9.2%).

Relevant toxicities were observed in about one third of the study population, including neurological toxicity in 31.7% (*n* = 38) of the sample and hematologic toxicity in 26.7% of cases (*n* = 32); the most common adverse effects were asthenia (73 cases; 60.8%) and emesis (70 cases; 58.3%). (Table 1).

**Table 1:** General characteristics of the study population (*n* = 120)*

	Overall
Age (yrs.)	49.3 (11.9)
BMI (kg/m^2^)	25 [22 – 29]
Waist (cm)	88 [81 – 98]
Hip (cm)	102 [96 – 110]
WHR	0.87 (0.07)
PA	5.3 [4.9 – 5.6]
FAT	32.7 (8.5)
FFM	67.5 [60.5 – 73.7]
ER	70 [0 – 90]
PGR	4 [0 – 60]
Ki67 antigen	55 [40 – 70]
Cancer type	
* B (Luminal B/HER2 negative)*	44 (36.7)
* BH (Luminal B/HER2 positive)*	34 (28.3)
* H (Non-luminal HER 2 positive)*	11 (9.2)
* Triple Negative*	31 (25.8)
Menopause	54 (45)
Therapeutic Scheme	
* Ec* + *12 Taxolo* ± *Herc OR 4EC1 ( Th1)*	95 (69.2)
* Ec* + *Carbo Taxolo OR Carbo tax* + *EC (Th2)*	25 (20.8)
Standard dose	102 (85)
Dose reduction	12 (10)
Postponement	14 (11.7)
Hypertransaminasemia	14 (11.7)
Hot flashes	5 (4.2)
Headache	7 (5.8)
Constipation	19 (15.8)
Epigastralgia	22 (18.3)
Hematologic toxicity	32 (26.7)
Neurological toxicity	38 (31.7)
Onychopathy	15 (12.5)
Osteoarticular pain	33 (27.5)
Asthenia	73 (60.8)
Diarrhea	16 (13.3)
Emesis	70 (58.3)
Mucositis	28 (23.3)
Reduction during	25 (20.8)
CHT interruption due to toxicity	9 (7.5)
CHT interruption due to non-response/progression	4 (3.3)

*BMI* Body Mass Index*, WHR* Waist–Hip Ratio*, PA* Phase Angle*, FAT* percentage of fats*, FFM* Fat-Free Mass*, ER* Estrogen Receptor*, PGR* Progesterone Receptor*, CHT* chemotherapy

*Descriptive statistics are expressed as median [interquartile range] or mean (standard deviation) for quantitative variables, as absolute and percentage frequencies for qualitative variables

### Toxicity, BMI, and body composition

We thus focused on the relationship between chemo-related toxicities, the different drugs combinations, BMI, and body composition.

According to the ordinary logistic regression models, a lower FM emerged as a protective factor against neurological toxicity (OR 0.95, 95%CI 0.91–1.00; *p* = 0.058), while no other body composition parameter did significantly affect the onset of hematologic toxicity (Table 2).

**Table 2:** Relationship between Toxicity and Body composition (*n* = 120)

	Ordinary Logistic Regression Model	Direct effect of Body composition	Predictor x Therapy (ref: Th1)
	Hematologic toxicity		
	OR (95% CI); *p*	OR (95% CI); *p*	IOR (95% CI); *p*
BMI	0.97 (0.89; 1.06); 0.540	1.03 (0.93; 1.14); 0.523	*Th*_*2*_* 0.49 (0.24; 1.01); 0.052* Th_3_ 0.98 (0.78; 1.22); 0.837
Waist	0.98 (0.95; 1.02); 0.416	1.00 (0.96; 1.05); 0.891	*Th*_*2*_* 0.89 (0.78; 1.02); 0.091* Th_3_ 1.02 (0.92; 1.12); 0.756
Hip	0.99 (0.96; 1.03); 0.700	1.01 (0.96; 1.06); 0.670	*Th*_*2*_* 0.86 (0.72; 1.03); 0.098* Th_3_ 1.02 (0.93; 1.13); 0.634
WHR	0.04 (0.00; 32.81); 0.350	0.11 (0.00; Inf^); 0.647	Th_2_ 0.00 (Inf^; Inf^); 0.412 Th_3_ 0.73 (Inf^; Inf^); 0.966
PA	0.98 (0.48; 2.03); 0.964	1.17 (0.45; 3.03); 0.754	Th_2_ 1.04 (0.10; 10.74); 0.974 Th_3_ 0.63 (0.14; 2.81); 0.544
FAT %	0.99 (0.94; 1.04); 0.689	1.03 (0.96; 1.10); 0.413	Th_2_ 0.82 (0.67; 0.99); 0.041 Th_3_ 1.00 (0.88; 1.14); 0.963
FFM %	1.01 (0.97; 1.06); 0.569	0.98 (0.93; 1.04); 0.628	Th_2_ 1.21 (0.99; 1.46); 0.056 Th_3_ 0.98 (0.87; 1.12); 0.810
	Neurological Toxicity		
BMI	0.94 (0.87; 1.03), 0.180	0.97 (0.89; 1.07); 0.571	Th_2_ 0.90 (0.66; 1.24); 0.530 Th_3_ 0.78 (0.56; 1.09); 0.139
Waist	0.99 (0.95; 1.02); 0.447	1.00 (0.96; 1.04); 0.957	Th_2_ 0.96 (0.86; 1.07); 0.467 Th_3_ 0.93 (0.84; 1.04); 0.202
Hip	0.97 (0.94; 1.01); 0.163	0.99 (0.95; 1.03); 0.631	Th_2_ 0.95 (0.82; 1.09); 0.462 Th_3_ 0.90 (0.80; 1.03); 0.123
WHR	13.26 (0.03; 6153.74); 0.409	37.24 (0.02; Inf^); 0.349	Th_2_ 0.02 (Inf^; Inf^); 0.684 Th_3_ 1.19 (Inf^; Inf^); 0.981
PA	1.50 (0.75; 2.99); 0.254	1.71 (0.73; 3.97); 0.212	Th_2_ 1.02 (0.75; 1.01); 0.988 Th_3_ 0.58 (0.13; 2.52); 0.467
FAT %	0.95 (0.91; 1.00); 0.058	0.97 (0.91; 1.03); 0.308	Th_2_ 0.92 (0.78; 1.10); 0.375 Th_3_ 0.91 (0.78; 1.06); 0.225
FFM %	1.05 (1.00; 1.10); 0.048	1.03 (0.98; 1.09); 0.254	Th_2_ 1.10 (0.91; 1.31); 0.323 Th_3_ 1.10 (0.94; 1.27); 0.230

*BMI* Body Mass Index*, WHR* Waist–Hip Ratio*, PA* Phase Angle*, FAT* percentage of fats*, FFM* Fat-Free Mass*, OR* Odds ratio*, 95%CI 95%* Confidence Interval*, Ref* Reference*, IOR* Interaction Odds Ratio*,*

*All models are age-adjusted. Ref: Th1

Looking at therapeutic protocols, in Th2 regimens a reduced BMI (IOR 0.49, 95%CI 0.24–1.01; *p* = 0.052), as well as a reduced waist and hip circumference emerged as a protective factor against hematologic toxicity (IOR 0.89, 95%CI 0.78–1.02; *p* = 0.091 and IOR 0.86, 95%CI 0.72–1.03; *p* = 0.098, respectively).

Moreover, in Th2 protocols, a suggestive association was found toward a negative role of a low FFM % (IOR 1.21, 95%CI 0.99–1.46, *p* = 0.056) in hematologic toxicity, as well as a significant protective role of a reduced FM% (IOR 0.82, 95%CI 0.67–0.99; *p* = 0.041).

No significant association emerged toward a role of body composition and the interruption of chemotherapy, due either to toxicity or to non-response/tumor progression (Table 3).

**Table 3:** Relationship between chemo interruption due to Toxicity and fat component (*n* = 120)

	Ordinary Logistic Regression Model	Direct effect of FAT composition	Predictor x Therapy (ref: Th1)
	Interruption due to Toxicity		
	OR (95% CI); *p*	OR (95% CI); *p*	IOR (95% CI); *p*
FAT %	0.94 (0.82; 1.07); 0.594	0.97 (0.87; 1.08); 0.566	Th_2_ 1.00 (Inf^; Inf^); 1.000 Th_3_ 1.05 (0.87; 1.27); 0.599
FFM %	1.02 (0.94; 1.11); 0.588	1.03 (0.93; 1.15); 0.559	Th_2_ 0.99 (Inf^; Inf^); 1.000 Th_3_ 0.98 (0.78; 1.15); 0.596
	Interruption due to non-response/progression		
FAT %	0.98 (0.90; 1.06); 0.362	0.90 (0.77; 1.06); 0.203	Th_2_ 1.13 (Inf^; Inf^); 1.000 Th_3_ 1.18 (0.85; 1.63); 0.328
FFM %	1.06 (0.93; 1.22); 0.364	1.11 (0.94; 1.31); 0.208	Th_2_ 0.88 (Inf^; Inf^); 1.000 Th_3_ 0.85 (0.61; 1.18); 0.333

*FAT* percentage of fats*, FFM* Fat-Free Mass*, OR* Odds ratio*, 95%CI: 95%* Confidence Interval*, Ref.* Reference*, IOR* Interaction Odds Ratio*, GLM* Generalized Linear Model

*All models are age-adjusted. Ref: Th1

### Body weight, body composition, adverse events, and tumor response

Among the most common chemo-related side effects, assessed through a clinical interview performed by oncologists before every cycle of chemotherapy and recorded in the patient’s reports, a suggestive association emerged for a protective role of a lower FM composition (OR 0.90, 95%CI 0.79–1.02; *p* = 0.091) against hot flashes, while a lower FFM% seems to promote their onset during chemotherapy (OR 1.11, 95%CI 0.98–1.26; *p* = 0.093). No other association between body composition and any other adverse event emerged (Table 4).

**Table 4:** Relationship between adverse events and FAT% and FFM% in the study population (*n* = 120)

	OR (95% CI); *p*	OR (95% CI); *p*	OR (95% CI); *p*	OR (95% CI); *p*
	Hot flashes	Headache	Constipation	Epigastralgia
FAT %	*0.90 (0.79; 1.02); 0.091*	1.00 (0.91; 1.10); 0.994	0.99 (0.93; 1.05); 0.666	1.00 (0.95; 1.06); 0.904
FFM %	*1.11 (0.98; 1.26); 0.093*	1.00 (0.92; 1.09); 0.931	1.02 (0.96; 1.07); 0.601	1.00 (0.95; 1.05); 0.976
	Onychopathy	Osteoarticular pain	Asthenia	Diarrhea
FAT %	0.95 (0.89; 1.02); 0.160	0.99 (0.94; 1.04); 0.692	0.99 (0.95; 1.04); 0.667	1.04 (0.98; 1.11); 0.209
FFM %	1.05 (0.98; 1.12); 0.156	1.01 (0.97; 1.06); 0.569	1.00 (0.96; 1.05); 0.878	0.97 (0.92; 1.03); 0.335
	Emesis	Mucositis		
FAT %	1.00 (0.95; 1.04); 0.865	1.01 (0.96; 1.06); 0.775		
FFM %	1.00 (0.96; 1.04); 0.961	0.98 (0.94; 1.03); 0.426		

*FAT* percentage of fats*, FFM* Fat-Free Mass*; OR* Odds ratio*, 95%CI 95%* Confidence Interval*, Ref.* Reference

*In bold the significant results (*p* < 0.05), in italic the suggestive results (0.05 < *p* < 0.10)

**All models are age-adjusted. Ref: Grade 1

More importantly, on ordinary logistic regression models and looking at interaction with therapies, no significant correlation was found between body weight, body composition, and pathologic response to drugs (Table 5).

**Table 5:** Relationship between pathologic complete response (pCR) and Body composition (*n* = 120)

			Ordinary Logistic Regression Model	Direct effect of Body weight/ composition	Predictor x Therapy (ref: Th1)
	pCR (*n* = 56)	npCR (*n* = 64)	OR (95% CI); *p*	OR (95% CI); *p*	IOR (95% CI); *p*
BMI	23.6 (20.9–29.6)	25.3 (22.7–28.2)	0.98 (0.92; 1.06); 0.671	1.00 (0.92; 1.09); 0.984	Th_2_ 0.99 (0.79; 1.26); 0.9671 Th_3_ 0.94 (0.76; 1.16); 0.561
Waist	87 (81–100.5)	88.5 (81–96)	1.00 (0.97; 1.03); 0.980	1.01 (0.97; 1.05); 0.515	Th_2_ 0.97 (0.89; 1.06); 0.532 Th_3_ 0.97 (0.89; 1.06); 0.540
Hip	101.5 (94.4–112.3)	102.5 (98–109)	1.00 (0.97; 1.03); 0.969	1.01 (0.97; 1.05); 0.705	Th_2_ 0.99 (0.89; 1.10); 0.814 Th_3_ 0.98 (0.90; 1.08); 0.724
PA	5.3 (5.1–5.6)	5.3 (4.9–5.5)	1.45 (0.76; 2.77); 0.259	1.45 (0.65; 3.23); 0.362	Th_2_ 0.39 (0.04; 4.03); 0.428 Th_3_ 1.30 (0.31; 5.51); 0.717
FAT %	30.8 (22.3–40.1)	34.2 (29.7–37.8)	0.97 (0.93; 1.01); 0.193	0.98 (0.92; 1.03); 0.399	Th_2_ 0.99 (0.87; 1.13); 0.907 Th_3_ 0.97 (0.86; 1.10); 0.664
FFM %	69.2 (60–77.8)	66.2 (62.2–70.2)	1.02 (0.98; 1.06); 0.363	1.01 (0.96; 1.06); 0.663	Th_2_ 1.02 (0.90; 1.16); 0.781 Th_3_ 1.04 (0.92; 1.17); 0.526

*BMI* Body Mass Index*, PA* Phase Angle*, FAT* percentage of fats*, FFM* Fat-Free Mass*, OR* Odds ratio*, 95%CI 95%* Confidence Interval*, Ref.* Reference*, IOR* Interaction Odds Ratio

*In bold the significant results (*p* < 0.05), in italic the suggestive results (0.05 < *p* < 0.10)

**All models are age-adjusted. Ref: Th1 and None/Partial response

## Discussion

In breast cancer patients undergoing NAC, chemotherapy drugs are currently dosed based on body weight and predicted BSA. This method has some caveats and limitations as it may underestimate important features in body composition that can influence toxicities and eventually outcomes, in the obese population as well as in normal weight women [20].

Many studies have confirmed that overweight and obesity have a clear impact on toxicities and outcomes in these patients [21].

In a recent analysis of BC patients treated with adjuvant chemotherapy, obese people were significantly more likely to receive dose delays due to toxicity (33.3% vs 5.9%, p = 0.0068) than normal weight patients. (12) In a retrospective study by Liu and collon 273 BC patients receiving NAC, obesity was associated with worse event-free survival [22].

Nevertheless, according to the ASCO guidelines on appropriate systemic therapy dosing for obese adult patients, full weight-based dosing of cytotoxic chemotherapy should be offered regardless of obesity status, although the panel recognizes that the interactions between BoCo, toxicities, and clinical outcomes are to be better studied and characterized [17].

Less data are available about the correlation of abnormal distribution of fat mass and other body components (muscle mass, bone mass, and body water) with chemo-related toxicities in normal weight patients [23].

Shachar reported that higher fat mass and lower muscle mass were found to be associated with increased risks of toxicity-induced modifications of treatment in BC patients receiving chemotherapy [20], while in the study of Aleixo et al. sarcopenia was reported to be associated with toxicity rates and time-to-treatment failure in metastatic BC patients receiving taxane-based chemotherapy [24].

Prado et al. confirmed that sarcopenia is a significant predictor of toxicity and time to tumor progression in metastatic BC, even in normal weight patients [25].

Moreover, in a study investigating the correlations between CT-derived body composition parameters and toxicity in early BC patients receiving anthracycline- and taxane-based chemotherapy, lower skeletal muscle mass was significantly associated with hospitalizations and hematological and gastro-intestinal toxicities [9]. A recent study shows that personalizing paclitaxel dosing based on BoCo, by extending the drug infusion duration may reduce the peripheral neuropathy rates while maintaining systemic exposure in patients with low muscle mass, who have been demonstrated at higher risk for this kind of toxicity [26]. This evidence explains the number of studies that have recently appeared in the Literature on the importance of complementing body weight, body surface area, and morphometric parameters, in order to identify more appropriate drug dosing strategies that may minimize toxicity rates and improve treatment outcomes. Analysis of BoCo seems a more effective predictor of pharmacokinetics but unlike BMI, body composition requires instrumental measurements [27, 28]. The majority of studies in cancer patients have used either CT-defined skeletal muscle at the third lumbar vertebra (L3) level or dual-energy X-ray absorptiometry (DXA) CT-based analysis or DXA scan, while the use of BIA has been very limited, even though this tool is easy to use, less expensive and without any radiation-associated risk [14, 29–32].

Iwase et al. reported that patients diagnosed with an early BC and a higher amount of visceral adipose tissue measured by computed tomography in the upper abdominal area had significantly shorter distant disease-free survival, owing to increasing insulin resistance [33, 34]. In addition to visceral adipose tissue, a significantly reduced amount of skeletal muscle mass was also associated with higher overall mortality rates in non-metastatic BC patients and survivors [35, 36]. Cespedes Feliciano et al. showed that the relative dose intensity (RDI) was significantly reduced in non-metastatic BC patients receiving anthracycline- and taxane-based chemotherapy, when higher visceral and intramuscular adiposity were found at CT scans [37, 38]. In this scenario, our study adds some useful insights about the association between body composition, assessed by BIA, and some specific toxicities (namely hematological and neurological) that have a significant impact on RDI, the appropriate delivery of full doses of chemotherapy protocols and on patients’ quality of life. We highlighted that, regardless of BMI, a lower fat mass has a significant protective role against hematologic toxicity, as well as against neurological toxicities, in chemotherapy protocols containing platinum (Th2). As regards the FFM, a suggestive association toward a negative role of a low FFM % in hematologic toxicity was found in Th2 protocols.

The present study has some clear limitations, due mainly to the retrospective design and the limited number of patients, which does not allow to draw any definitive conclusion about the role of body composition on toxicity rates among women with the same BMI but different percentages of FM and FFM. Moreover, the main toxicities (gastro-intestinal, hematological, or neurological), potentially affecting dose reductions, postponement, or interruption, were assessed by oncologist, but patients were not given any specific questionnaire for side effects (such as hot flashes). As a result, the rates of side effects affecting the quality of life could be less accurate and potentially underrated. Despite this, the findings highlighted promising perspectives of a number of parameters other than BMI and morphometric measures, which could optimize the ways we dose chemotherapy drugs in clinical practice. The points of strength of the study include a very homogeneous population of patients enrolled, affected by the same type of cancer, in a narrow range of age, similar stages of the disease, and a balanced distribution of different subpopulations, according to the BMI and BC subtypes. It is worth mentioning also that the study has used the BIA procedure to assess body composition in this population of BC patients.

## Data Availability

No datasets were generated or analysed during the current study.

## Abbreviations

NAC: Neoadjuvant chemotherapy
BSA: Body surface area
BMI: Body mass index
FM: Fat mass
FFM: Fat-free mass
ASCO: American society of clinical oncology
WHR: Waist–hip ratio
Cm: Centimeter
SMF-BIA: Segmental multi-frequency-bioelectrical impedance analysis
Z: Resistance
Xc: Reactance
Φ: Phase angle
TBW: Total body water
Type B: Luminal B/HER2 negative
Type BH: Luminal B/HER2 positive
Type H: Her2 positive
TN: Triple negative
Th1: Anthracycline-taxane-based regimen
Th2: Anthracycline-taxanes plus carboplatin regimen
BIA: Bioelectrical impedance analysis
SD: Standard deviation
IQR: Median and interquartile range
ORs: Odds ratios
IOR: Interaction odd ratios
IRR: Interaction risk ratios
CHT: Chemotherapy
FAT: Percentage of fats
RR: Risk ratio
pCR: Pathologic complete response
DXA: Dual-energy X-ray absorptiometry
